# "Alexandra Day" for Cardiff

**Published:** 1913-05-10

**Authors:** 


### "Alexandra Day" for Cardiff.

It has been decided by a largely attended meeting of ladies and gentlemen convened by the Lord Mayor of Cardiff, Alderman Morgan Thomas, that Cardiff is to have its "Alexandra Day" on Tuesday, July 8, this year. The scheme was explained to the meeting by Mr. Rea, the secretary of King Edward VII.'s Hospital, to which institution the proceeds of the fête will be mainly devoted. It was announced that the Marchioness of Bute, the Countess of Plymouth, Lady Aberdare, and Lady Ninian Crichton-Stuart had consented to act as patronesses of the fête. The Lady Mayoress of Cardiff, Mrs. Morgan Thomas, was unanimously appointed president, with a strong representative executive committee. It was calculated that Cardiff should sell from 100,000 to 120,000 flowers, and the commemoration of "Alexandra Day" in association with "King Edward VII.'s Hospital" was regarded as peculiarly appropriate.

